# Effect of Germination on the Avenanthramide Content of Oats and Their in Vitro Antisensitivity Activities

**DOI:** 10.3390/molecules27196167

**Published:** 2022-09-20

**Authors:** Yuchao Feng, Decheng Suo, Xin Guan, Shi Wang, Zhiming Xiao, Yang Li, Xiaolu Liu, Xia Fan

**Affiliations:** 1 Institute of Quality Standards and Testing Technology for Agro-Products, Chinese Academy of Agricultural Science, Beijing 100081, China; 2 College of Food, Heilongjiang Bayi Agricultural University, Daqing 163319, China; 3 Chinese National Engineering Research Center, Daqing 163319, China

**Keywords:** avenanthramides, UPLC-QE HF HRMS, germination, oats, antiallergic

## Abstract

In this study, a method, based on an ultraperformance liquid chromatography coupled with high-field quadrupole orbitrap high-resolution mass spectrometry (UHPLC-QE-HF-HRMS) platform, was established for the trace determination of three major avenanthramides (AVNs). The MS conditions for determining the AVNs were optimized, and the cracking methods of avenanthramides were analyzed. The linear range of the results and the correlation coefficient were 1–2000 μg/L and >0.996, respectively. Further, the established method was employed for the determination of the AVN contents of oats at different germination times, and the results indicated that the AVN contents of Zaohua and Bayou oats increased 19.26 and 6.09 times, respectively, after germination. The total AVN content of both oat varieties reached a maximum on the fifth day of germination (153.51 ± 4.08 and 126.30 ± 3.33 μg/g for the Zaohua and Bayou oats, respectively). Furthermore, this study investigated the antiallergic and antioxidant activities of the germinated oats via hyaluronidase inhibition and 2,2-diphenyl-1-picrylhydrazyl (DPPH)-scavenging assays. The antiallergic and DPPH-scavenging abilities of the ungerminated forms of both oat varieties were weaker. However, on the fifth day of germination, the inhibition rate of anthranilamide hyaluronidase reached 72.7% and 67.3% for the Zaohua and Bayou oat varieties, respectively. The antiallergic abilities of the oats increased significantly on the fifth day of germination in terms of their antiallergic capacities and DPPH clearance (82.67% and 77.64% for the Zaohua and Bayou oats, respectively), and the two indicators exhibited similar trends. These findings demonstrated that AVNs exhibit good antisensitivity and antioxidation properties, and the antisensitivity effect correlated positively with the AVN content.

## 1. Introduction

**Figure 1:**
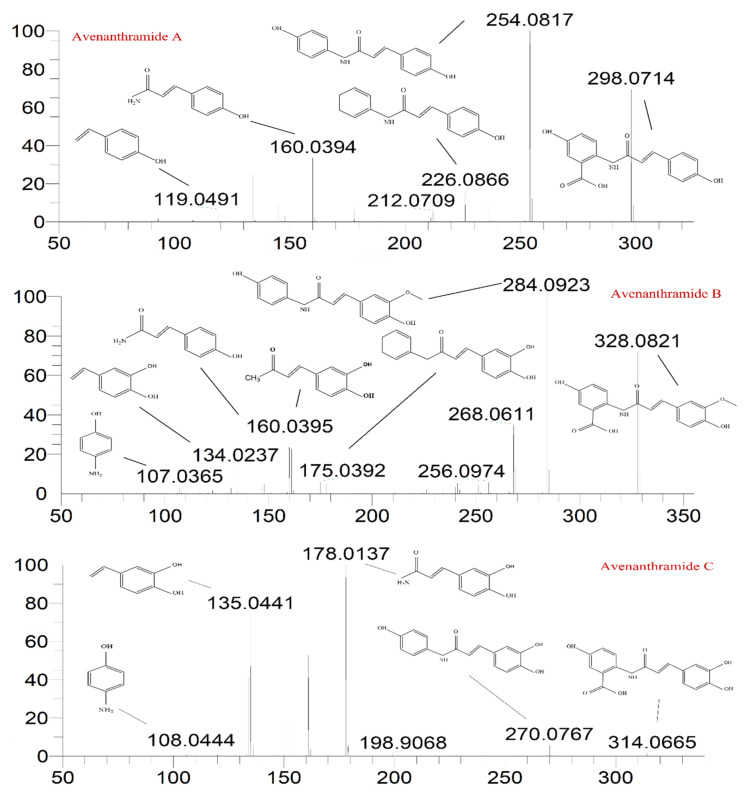
Mass spectra and theoretically possible fragmentation structures of the three AVNs.

Oats (*Avena sativa* L.) are among the most consumed whole grains worldwide [1], accounting for a long cultivation history. Oats are nutritional and functional, supplying energy and nutrition to the body while availing significant antioxidant, anticancer, anti-inflammatory, anti-obesity, and anti-atherosclerosis benefits, as well as regulating intestinal flora, improving insulin levels, lowering cholesterol levels, reducing the risk of cardiovascular disease, modulating the immune system, and ensuring many other health benefits [2]. Further, the functional properties of oats have been confirmed in cells, animals, and humans [3]. In addition to proteins, dietary fibers, and other major components, previous studies revealed that oats also contain a variety of trace phytochemicals, such as phenolic acids, flavonoids, and AVNs, exhibiting unique functionalities [3]. Among these trace phytochemicals, AVNs are unique low-molecular-weight soluble phenolic alkaloids that are only present in oats [4,5]. They are mainly present in oat seeds and are distributed in the bran, seed coat, and germ. The covalent bonding between hydroxyl cinnamic acid and o-aminobenzoic acid via an amide linkage [6] produces many AVNs in oats; among them, *N*-[4-hydroxy-(E)-cinnamoyl]-5-hydroxyanthranilic acid (AVN-A), *N*-[4-hydroxy-3-methoxy-(E)-cinnamoyl]-5-hydroxyanthranilic acid (AVN-B), and *N*-[3,4-dihydroxy-(E)-cinnamoyl]-5-hydroxyanthranilic acid (AVN-C) account for the three most abundant, and their structural formulae are shown in Figure 1 [7]. Thus far, over 30 AVN species have been identified [8], although most existing studies focused on AVN-A, AVN-B, and AVN-C. Presently, there are only three kinds of commercial standard substances for AVN; therefore, the amounts of the three AVNs listed above are generally employed to evaluate the overall AVN contents of oats. Regarding the quantification of other identified anthranilamides, most of them are “semi-quantitative” because of the unavailability of standards, i.e., their quantifications depend on the relative response of the structurally closest standard AVN to the internal standard.

The research on the qualitative and quantitative detections of AVNs in oats and oat products have increased gradually since the 1980s, and the corresponding detection platforms and methods have been continuously updated. However, some of these methods exhibited shortcomings. For example, high-performance liquid chromatography (HPLC) combined with ultraviolet diode-array detection (UV/DAD) [5] is limited by the challenge of fully separating the peak of each AVN and the impossibility of distinguishing the chemicals via their UV absorption spectra; these result in the inaccurate quantification of AVNs and their inapplicability at low concentrations. For HPLC coupled with electrochemical detection [9], although electrochemical detection is sensitive, it is only partially selective. Further, the liquid chromatography–tandem mass spectrometry (LC–MS/MS) method [10] facilitates the quantification of AVNs via ion monitoring, and it exhibits good sensitivity. Ishihara et al. [11] developed a multiple reaction monitoring (MRM) method based on LC–MS/MS to quantify three newly isolated AVNs; however, the method was not validated. Xie et al. [12] combined HPLC with triple quadrupole MS (TQMS) to determine three major AVNs, after which they employed an internal standard method to reduce the bias of the results. The method was sensitive, delivering nanogram-level limits of quantification and detection (LOQ and LOD, respectively); however, it still presented cases involving the simultaneous detections of other AVNs with the same qualitative ions in the oat samples. Considering that the complex composition of the matrixes of oat seeds or products can affect the detection results, other detection methods of AVNs with higher accuracy and a simpler pretreatment process must be explored.

Owing to their various biological activities, AVNs exhibit great application potential in food and related fields. Oats are the only viable dietary source of AVNs [13], although their amounts in oats are small and susceptible to factors, such as the variety [14], origin, and processing methods of the oat [13,15], so it is necessary to study the enrichment of avenanthramides. For example, germination represents a strategy for enriching bioactive substances and has been certified in other plant seeds [16,17]. Studies have also demonstrated that germination can increase the AVN content of oats [18], but the change in such AVN content during germination is unknown; thus, the analysis of the enrichment rule in the germination process will greatly benefit the development and application of AVNs. It is known that AVNs are strong antioxidants that exhibit significant anti-atherosclerotic efficacy [19]. Additionally, studies have demonstrated that AVNs can be employed to relieve skin inflammation and pruritus and that their structures are very similar to those of trinostat (*N*-3,4-dimethoxycinnamoyl o-aminobenzoic acid, Rizaben^®^), an antiallergic drug [20,21]. Dhakal et al.’s recent study [22] on the antiallergic inflammatory effects of AVN-C from germinated oats on mast cells revealed that it can be a candidate therapeutic agent for mast cell-mediated allergic inflammation. However, allergy is a very complex condition and the diversity of allergens causes a variation in the allergy types, and abundant research is still required to support this conclusion.

This study is aimed at establishing a rapid method for determining the three major AVNs in oats based on the ultra-HPLC coupled with high-field quadrupole orbitrap high-resolution MS (UHPLC-QE-HF-HRMS) platform and to resolve their cleavage modes. The effect of the germination treatment on the AVN content, as well as the differences between the compositions of the AVN contents of different varieties of oats, were also investigated. The in vitro antisensitizing activity and antioxidant properties of AVNs were also investigated employing hyaluronidase inhibition and 2,2-diphenyl-1-picrylhydrazyl (DPPH) free-radical-scavenging assays.

## 2. Results

### 2.1. Optimization Methods

The HRMS parameters of the three AVNs were optimized by injecting QE-HF-HRMS with a continuous microflow jet pump in the parallel reaction monitoring (PRM) mode of negative ionization (HEI^−^). The detection results obtained a high sensitivity. In this study, the capillary voltage and characteristic fragment ions were fully optimized, and the carbon-equivalent (CE) values were 10, 20, 30, 40, 50, and 60. The mass spectra are shown in Figure S1. At CE = 10, the parent ions of AVN-A and AVN-B were not separated. However, at CE = 50, the parent and large daughter ion fragments were dissociated into smaller ion fragments. The parent ion disappeared at CE = 60. Thus, CE values of 20, 30, and 40 exhibited better results, but CE = 30 and 40 exhibited the best results. Thus, the parent ion of AVN-C was dissociated at CE = 10, 20, and 30, and the same high-response intensity fragment ions were present, while all the parent ions disappeared at CE of ≥40. Therefore, combining the effects of the three AVNs, the optimized CE values were set to 20, 30, and 40.

This part of the information is correct. This part of the information is correct.QE HF HRMS indicated that the dissociation forms of the three AVNs were similar. The fragment ion, *m*/*z* 254.0817, of the excimer ion, *m*/*z* 298.0714, of AVN-A decreased by 44 Da, and molecular decarboxylation proceeded on the left side of the m-hydroxybenzoic acid. The fragment ion at *m*/*z* 226.0866 decreased by 28 Da from *m*/*z* 254.0817, following the removals of carbon and oxygen at the left benzene ring that was attached to the hydroxyl group on the left side and rearrangement of the ring. The fragment ion at *m*/*z* 160.0394 was based on *m*/*z* 226.0866, dissociated from the amino group, and stripped of the left-ring structure, while the fragment ion at *m*/*z* 119.0419 was based on *m*/*z* 160.0394 and stripped of the amino and carboxyl groups. The excimer ion of AVN-B was *m*/*z* 328.0821, and the fragment ions at *m*/*z* 284.0923 and 256.0974 were cleaved in the same manner, as that in AVN-A, decreasing by 44 and 28 Da, respectively. The fragment ion at *m*/*z* 175.0392 was dissociated between the amino group and carbon–oxygen double bond to remove the ring structure on the left side based on *m*/*z* 256.0974. The fragment ion at *m*/*z* 160.0395 was dissociated from the left side between the amino group and benzene ring under the original molecular structure condition to remove the m-hydroxybenzoic acid, and a hydroxyl group was removed from the right-side benzene structure. The fragment ion at *m*/*z* 134.0237 is a small-molecule structure that dissociated from the carbonyl group and double bonds in the original molecular structure. The fragment ion at *m*/*z* 107.0365 corresponded to the aminophenol structure. The excimer ion of AVN-C was *m*/*z* 314.0665, and the fragment ion at *m*/*z* 270.0767 was the same as the break site of AVN-A and AVN-B, decreasing to 44 Da. The fragment ion at *m*/*z* 178.0137 was dissociated from between the amino and benzene rings on the left side in the original molecular structure, after which the m-hydroxybenzoic acid structure was removed. The fragment ions at *m*/*z* 135.0441 and 108.0444 were identical to the two small fragment ions of AVN-B. Based on the cleavage pattern of HRMS, its analogs, derivatives, and metabolites could also be readily identified, thus offering a strong theoretical basis for their quantitative analysis. The mass spectral conditions of the three AVNs, which were obtained via this method, are listed in Table 1. The final fragment ions, *m*/*z* 254.0817, 284.0923, and 178.0137, were selected for AVN-A, AVN-B, and AVN-C, respectively, as qualitative ions.

**Figure 2:**
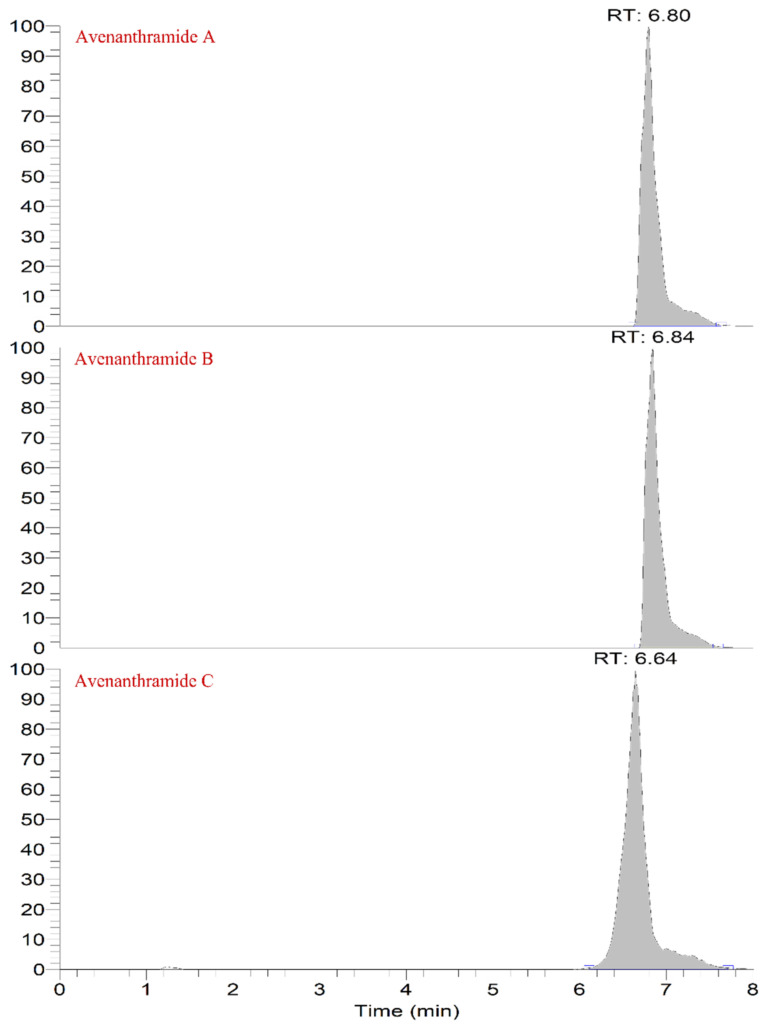
Chromatograms of the three anthraceneamides.

As the instrument is a combination of the parent ion selectivity of a high-performance quadrupole and the high-resolution accurate mass number (HR/AM) orbitrap detection technique, it exhibits excellent performance, and the MS conditions are crucial to the establishment of the method. Further, the LC conditions, including the flow rate, column temperature, and elution gradient, were also optimized. The flow rates were set as 0.2, 0.25, 0.3, 0.35, 0.4, and 0.45; it was observed that the higher the flow rate, the shorter the peak time and the stronger the response intensity of the AVN specimen. Therefore, the flow rate was set as 0.45, and the column temperatures were set as 25, 30, 35, and 40 °C, respectively. The elution gradients were first set to 1.5, 2.5, 3.5, and 4.5 min to vary them. Thus, the intensity of the AVN response and the retention time increased gradually. The first elution gradient was set to 3.5 min considering the detection time and corresponding intensity. The optimal liquid-phase conditions for quantifying the three major AVNs are listed in Table 2, and Figure 2 shows the chromatograms of the three AVNs under the optimum conditions; moreover, the three AVNs were detected within 8 min. The gradients, as set by the standard curves of the three AVNs, were detected under the optimum conditions, and the results are presented in Table 3.

Recovery methods and precision test. To investigate the accuracy and reproducibility of the method, the Bayou variety of the oat samples, which germinated for one day, was randomly selected as the matrix for the spiked recovery test. Samples with different contents were prepared. Further, six parallel tests were conducted with the same 1 content in each batch, and the three contents were set at 0.5, 1, and 1.5 μg/kg, respectively. Table 4 indicates that the recoveries of the three types of AVNs are 100.4–118.0%, which correlates with the recovery range of a certain content. The coefficient of variation (CV, %) was ≤20%; however, the oat samples exerted a certain matrix-enhancement effect, indicating that the established method exhibited good accuracy and repeatability.

### 2.2. Variation Pattern of the AVN Content of the Germinated Oats and the Effect of Variety on the Content

In this study, two varieties of oats were selected and germinated separately for 0–8 days, with ~10 cm sprouts on the 8th day. A separately established method was employed to detect the AVN content of the sprouted oats, and the results are shown in Table 5. The total content of the three types of AVNs in the Zaohua and Bayou oat varieties tended to increase first before decreasing. On the 5th day of germination, the maximum AVN contents, 153.51 ± 4.08 and 126.30 ± 3.33 μg/g, were detected in the Zaohua and Bayou oats, respectively. After germination, the AVN contents of the Zaohua and Bayou oats were 19.26 and 6.90 times higher than that of the ungerminated oat, respectively. Without the germination treatment, the total content of the three main AVNs in the Bayou oat varieties was high, 2.29 times that in the Zaohua varieties. Table 3 reveals that the enrichment degree of the AVNs in the Zaohua oat varieties was higher than that in the Bayou varieties after the optimum germination time treatment, and the AVN content of the Zaohua varieties was 1.21 times higher than that of the Bayou varieties.

The AVN-A and AVN-B contents of early flowering oats first increased before decreasing, reaching a maximum on the fifth day. AVN-C exhibited an upward trend and reached its maximum content on the eighth day. The contents of the three types of AVNs in the ungerminated oats (the samples treated for 0 days) were in the following order: AVN-C > AVN-A > AVN-B, and their maximum contents were in the following order: AVN-C > AVN-B > AVN-A after germination. The three types of AVNs in the Bayou and Zaohua oats exhibited the same variation trends. The AVN-A and AVN-B contents first increased, after which they decreased, reaching a maximum on the fifth day. AVN-C displayed an upward trend and reached its maximum content on the eighth day. However, the order of their contents in the ungerminated oats was AVN-A > AVN-B > AVN-C, and that of the highest content, following the germination treatment, was AVN-B > AVN-C > AVN-A. Finally, the contents of the three types of AVNs in the different oat varieties were different, and they can be significantly increased via the germination treatment; moreover, the enrichment degrees of AVN-C and AVN-B are higher than that of AVN-A. 

### 2.3. In Vitro Antisensitivity and Antioxidant Activities of the Germinated Oats

**Figure 3:**
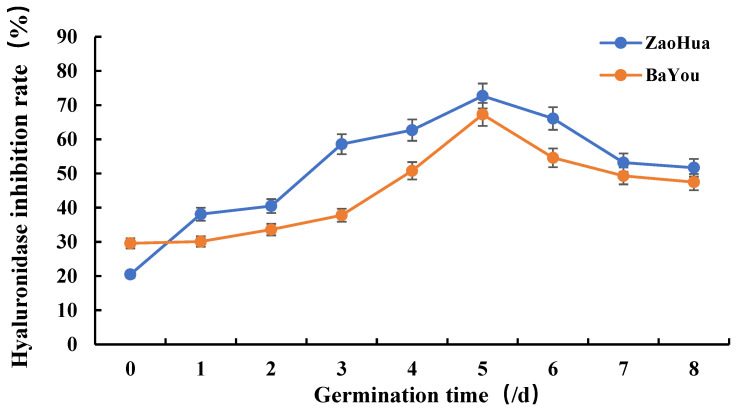
In vitro hyaluronidase-inhibition activities of the anthracamide (AVN) extract of the oats at different germination times.

The inhibition rate of hyaluronidase is a reflection of the antiallergic activity of the sample. In the extant studies, the in vitro hyaluronidase inhibition test has generally been conducted to explore the anti-allergy activities of the samples, and this represents a crucial indicator in the development of antiallergic drugs [23,24]. Figure 3 shows the in vitro hyaluronidase-inhibition rates of the two oat varieties at different germination times. The figure shows that the hyaluronidase-inhibition rate of the AVNs in the two oat varieties first increased before decreasing at different germination times, reaching the maximum value on the fifth day. This variation trend is the same as that of the total content of the three types of AVNs. The hyaluronic acid inhibition rates of ungerminated Zaohua oat and Bayou Oat were 20.5% and 29.6%, respectively, showing weak anti-allergy ability. However, the inhibition rates increased to 72.7% and 67.3% on the fifth day of germination, respectively. Further, the Zaohua oat variety was more resistant than the Bayou one. In summary, the findings confirmed that AVN exhibited good hyaluronidase inhibitory activities, i.e., it exhibits a good anti-allergy ability and a dose–effect relationship. Furthermore, germination can greatly improve the antisensitization ability of oats. Oats are a hypoallergenic food, and some of their unique AVN constituents exhibit antiallergenic activities. Thus, the antiallergic activity and antioxidant activity may exhibit a certain internal relationship, but this is yet to be explored.

**Figure 4:**
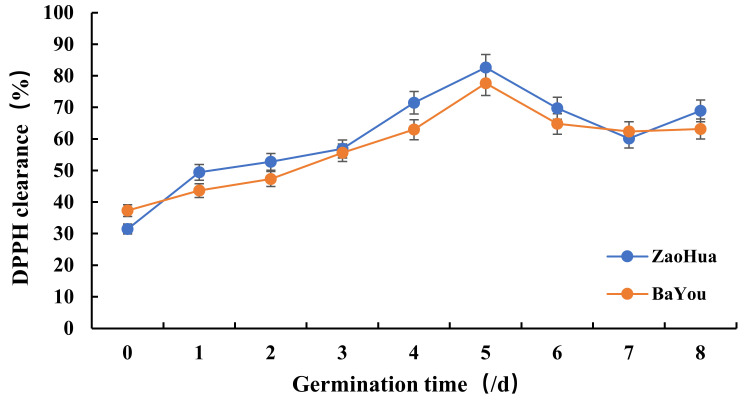
DPPH free-radical-scavenging rate of the AVN extract of the oats at different germination times.

Studies have demonstrated the close relationship between antiallergic and antioxidant activities, and a good scavenging effect of oxygen free radicals is key to alleviating allergies [25]. AVN is a potent antioxidant. Here, the DPPH free-radical-scavenging rates of the AVN extracts were measured at different germination times, and the results are shown in Figure 4. The AVN extracts of the two oats first increased, after which they decreased with increasing germination time. On the fifth day, the DPPH free-radical-scavenging rates reached the maximum; the maximum clearance rates of the AVN extracts of the Zaohua and Bayou oat varieties were 82.67% and 77.64%, respectively. Both varieties exhibited strong antioxidant activities, and these results are consistent with the results of the AVN content and hyaluronidase-inhibition rate. These results demonstrated that the AVN content exhibited a dose–effect relationship with its sensitivity and antioxidant activities. Concurrently, a certain internal relationship exists between antisensitivity and antioxidant activities.

## 3. Discussion

Compared with existing methods for detecting AVNs, the established method differed in the fragmentation mode of the parent ions that were selected in the qualitative ions and MS, and this was closely related to the type of instrument. Regarding the detection accuracy, the LODs and LOQs were low, thus enabling the detection of low concentrations of AVNs. When calculating the AVN content employing the integration system of the instrument, the qualitative chromatographic peak was significant, and a few miscellaneous peaks appeared, indicating that the established detection method was barely affected by the matrix interference and exhibited good specificity, and these enhanced the detection accuracy. This method can also be employed to determine the AVN contents of other oat samples. During the extraction of AVN, an 80% ethanol solution, which is simpler and more conducive than those employed in other studies, was utilized as the extraction solution to prepare the acidic liquid-phase extraction system. Concurrently, employing ultrasonic-assisted extraction, repeating the extraction process two times can ensure the complete extraction of AVNs in oats. This method is simple and fast for the extraction and detection of anthracene.

The varieties of germinated cereals have increased in recent years, and the nutritional and bioactive components of cereals increase after germination. Additionally, the physical and chemical properties of foods produced from sprouted grains can be improved [26,27], and this is true for germinated oats. Krapf et al. germinated oats for three days and observed that the reduction in the sugar content increased the α-amylase activity, and this is closely related to the germination degree [28]. Tian et al. reported that the vitamin and total polyphenol contents of oats increased significantly after three days of germination [29]. Xu soaked and germinated oats, observing that their phenolic acid content increased significantly [30]. AVN, as a polyphenolic alkaloid, can also be detected in the germination process. Damazo-Lima et al. [31] germinated oats for five days and observed that their polyphenol contents increased. Ultrapure LC (UPLC)/MS was employed to analyze the methanol extract of germinated oats, and the detected AVNs were AVN-D, AVN-L, and AVN-G/1c/2P, among which the AVN-D content was highest. Notably, germination can increase the AVN content, as demonstrated by the results of this study. However, existing related studies did not explain the basis of the selected germination days, and only a few studies exist on the change rule of the active-substance content of oats during germination. This study can provide a certain basis.

Sprouted oats with green leaves do not qualify as sprouted whole grains, because they exceed the standard. However, this study still set the germination time to eight days to explore the change rule of the AVN content of oat during the germination process, rather than simply preparing germinated cereal food. AVNs have exhibited many functions, and they are a potential bioactive substance. However, it is necessary to explore methods for improving the AVN contents of oats, which are very low. Germination positively affects the enrichment of active substances. The preparation of oats as germinated whole grain food gradually increases their AVN contents, thereby improving their bioactivities. By germinating the oats via the three main enrichment methods, Table 3 reveals that the AVN-A and AVN-B contents were highest on the fifth day of germination and that these contents decreased afterward. The enrichment time node was on the fifth day of germination. The AVN-C content increased with increasing germination time. Regarding the germination days in this study, the highest content was observed on the eighth day. Moreover, the optimal enrichment time node could be further studied if AVN-C was enriched.

Studies on the antioxidant activities of oat grains, as well as the three AVNs, have been reported [32]. For example, the antioxidant activities of the three main AVNs followed this order: AVN-C > AVN-B > AVN-A. In this study, the antioxidant capacities of the AVN extract of oats were evaluated at different germination times, employing the DPPH free-radical-scavenging rate. Compared with the determination of the antioxidant activities of oat grains, this method was more accurate, because it could exclude the interference of other components in oats. Compared with the determination of a single AVN, it was more beneficial to determine the rule of the change in the antioxidant activity of AVN during the germination of oats. To study the antisensitivity activity of AVN, the hyaluronidase-inhibition experiment was adopted as the in vitro method, and the rest were in vivo experiments in animals. In this study, changes in the antisensitivity activity of AVN were investigated at different germination times, and the antioxidant activity was determined. Notably, AVN exhibited antisensitivity, and its antioxidation activity and the antisensitivity effect correlated positively with the AVM content.

## 4. Materials and Methods

### 4.1. Reagents and Materials

The oat seeds were of 2021 output and Zaohua 1 and Bayou 1 varieties. Both were naked oat species. Their granules were full and free of pests and diseases. They were purchased from Yimu Agricultural Technology Co., Ltd. (Zhangjiakou, China).

Methanol, acetonitrile, and formic acid were of chromatographic grade. They were purchased from Fisher ChemAlert Guide (Waltham, MA, USA). Primary water was utilized as the experimental water. Hyaluronidase was purchased from Solarbio Science & Technology Co., Ltd. (Beijing, China); AVN-A, oat AVN-B, and oat AVN-C standards (purity, ≥98%) were purchased from Rongan Biotechnology Co., Ltd. (Xi’an, China).

### 4.2. Instruments and Equipment

A Q Exactive HF mass spectrometer, a Heraeus Fresco Centrifuge (Thermo Fisher Scientific, Waltham, MA, USA), an Intelligent sample grinder (Beijing Newpark Biotechnology Co., Ltd., Beijing, China), BSA124S-CW Scales (Sartorius), a PS-60AL ultrasonic cleaner (Shenzhen Redbone Electronics Co., Ltd., Shenzhen, China), and a TU-1800 UV–visible spectrophotometer (Beijing Purkinje General Instrument Co., Ltd., Beijing, China) were utilized for the experiments.

### 4.3. Establishment of the Anthranilamide Assay

#### 4.3.1. Preparation of the AVN Standard

Appropriate amounts of the AVN-A, AVN-B, and AVN-C standards were dissolved in dimethyl sulfoxide and configured into a standard stock solution with a concentration of 1 mg/mL. Next, 1 mL of each of the three standard reserve liquids was removed and placed in a 100 mL volumetric flask. Thereafter, these reserve liquids were configured into a 10 μg/mL standard intermediate mixture. This intermediate solution was further diluted into solutions with concentrations of 1, 10, 50, 100, 250, 500, 1000, and 2000 ng/mL, after which the standard curve was drawn with the standard concentration and peak area as the abscissa and ordinate, respectively. Among them, 1000 ng/mL of the mixed standard solution was utilized to establish the method.

#### 4.3.2. Method Establishment

Instrument platform: A Q Exactive HF spectrometer, column: waters column: BEH C18, 1.7 μm/3.00 × 100 mm. Based on the nontargeted assay that was established in a previous study, three anthranilamide mixed standards were first tested. Full MS/dd-MS 2 (top-n) was employed for the detection of anthranilamide in the positive/negative ion mode, and the anthranilamide substances were detected in the negative ion mode. Chromatographic conditions: mobile phase A in liquid chromatography was 1% formic acid water, mobile phase B was acetonitrile, and the temperature of the sample tray was 4 °C. The MS conditions: electrospray voltage, 3.5 kV; sheath gas, 30 Arb; auxiliary gas, 20 Arb; ion transport tube temperature, 350 °C; auxiliary gas heater temperature, 300 °C; full MS resolution, 120,000; MS/MS resolution, 60,000.

The scanning mode of QE-HF-HRMS was changed into negative ionization (HEI^−^) and PRM modes after the detection of the three anthranilamide samples. The chromatographic conditions were kept constant. The MS conditions were as follows: electrospray voltage, 3.5 kV; sheath gas, 30 Arb; auxiliary gas, 20 Arb; ion transfer tube temperature, 350 °C; auxiliary gas heater temperature, 300 °C; MS/MS resolution, 60,000. The targets were determined according to the molecular weights of the three anthranilamide species.

Finally, the main influencing factors, namely the collision energy (CE), column temperature, flow rate, and chromatographic gradient, were optimized, while other conditions were automatically optimized by the instrument.

### 4.4. Germination Treatment of the Oats

Following Lima et al.’s [31] method, appropriate amounts of the oats were first weighed and soaked in a 1.5% sodium hypochlorite solution (1:6/*w*:*v*) for 30 min at 25 °C. Afterward, the soaking liquid was poured off, after which the oats were repeatedly rinsed with distilled water to a pH of ~7.0. Next, the oats were added to distilled water (1:6/*w*:*v*) and sonicated for 5 min at 60 w. After 4 h of soaking, the oats were removed and drained in a tray, after which they were wrapped in a soaked towel and stored in a biochemical incubator at 20 °C and relative humidity of >80% for the germination. Some of the samples were collected every 24 h. Thus, eight samples were collected consecutively in three parallel groups each time and recorded. Both oat variety samples were treated by the same procedures.

### 4.5. Extraction of Anthranilamide

Following Xie’s [12] and Jastrebova et al.’s [10] methods, the germinated oat samples were oven-dried at 40 °C to a constant weight. Thereafter, they were crushed by an electric grinder and allowed to pass through a 70-mesh sieve. The sample was defatted by petroleum ether for 5 h. Afterward, 0.5 g of the defatted oat powder was weighed, and 5 mL of an 80% ethanol solution was added, after which the mixture was vortexed, mixed for 5 s, and sonicated for 60 min in an ultrasonic cleaner at 150 W and 45 °C. Thereafter, the mixture was centrifuged for 5 min at 10,000 r, after which the supernatant was collected. The precipitate was further mixed with 5 mL of the 80% ethanol solution and centrifuged. The two supernatants were combined and passed through a 0.25 μm filter membrane for the determination of the anthranilamide content via the method established in Section 4.3.2.

### 4.6. In Vitro Determination of the Anti-Allergy

Oat amide extracts with different germination times were prepared for the hyaluronidase-inhibition assay, i.e., the sample solution, as described in Section 4.5. Following the Elson–Morgan method, the sample solution (0.5 mL) and 0.5 mL of hyaluronidase (500 unit/mL) were mixed for 20 min at 37 °C. Next, 2.5 mol/L of calcium chloride (0.1 mL) was added and maintained for 20 min at 37 °C. Afterward, potassium hyaluronate (0.5 mg/mL) was added and maintained for 40 min at 37 °C. Thereafter, the mixture was maintained for 5 min at room temperature. Finally, two drops of 5 mol/L of NaOH and 0.5 mL of an acetylacetone solution were added and heated in boiling water for 15 min. Next, the solution was immediately cooled in ice water for 5 min and incubated at room temperature for 10 min. Thereafter, 0.5 mL of water and 0.5 mL of Ehrlich and anhydrous ethanol were added to a constant volume of 10 mL. After shaking and mixing, the absorbance was measured at 530 nm by the following equation:

$$R=\frac{\left(B-A\right)-\left(D-C\right)}{B-A)}\times 100\%$$

where *R* is the hyaluronidase-inhibition rate, *A* is the control blank ABS value (with an acetate buffer solution rather than the sample solution), *B* is the control solution ABS value (with an acetate buffer solution rather than the sample solution and an enzyme solution), *C* is the specimen solution ABS value, and *D* is the absorbance value of the specimen solution (with an acetate buffer solution rather than an enzyme solution). In the experiment, the full wavelength scan of group A specimens (wavelength range = 450–700 nm) was employed to determine the maximum absorption wavelength, with water as a reference. The absorbance values were measured at the maximum absorption wavelength for the other specimens.

### 4.7. Measurement of the DPPH Free-Radical-Scavenging Rate

The oat amide extracts with different germination times were prepared for the *DPPH* scavenging assay, i.e., the solution to be tested, as described in Section 4.5. Here, 3 mL of a *DPPH* working solution was added to 1 mL of the solution to be measured (the absorbance of the *DPPH* working solution was 0.70 ± 0.02 at that time), shaken well, and maintained in the dark for 30 min. Anhydrous ethanol was selected as the blank reference, and an absorbance value (*A1*) was measured at 515 nm. The anhydrous ethanol (1 mL) was added to 3 mL of the DPPH working solution, and the absorbance value (*A2*) was measured under the same conditions. Further, 1 mL of the solution to be measured was added to 3 mL of the anhydrous ethanol, and the absorbance value (*A3*) was measured under the same conditions. The DPPH radical-scavenging rate was calculated, as follows:

$$DPPH\left(\%\right)=(1-\frac{A1-A3}{A2})\times 100\%$$

### 4.8. Data Analysis

The experimental analysis results were presented as average values, and the experimental results were plotted with Microsoft Excel (2021).

## 5. Conclusions

Based on the UHPLC-QE-HF-HRMS platform, a rapid method was established for the detection of the three main AVNs in oats. The method exhibited high sensitivity and good accuracy and can be applied to the detection of AVN in oats and its related products, exhibiting complex matrices. The results demonstrated that the germination treatment significantly increased the anthracene contents of the oats, highlighting a good strategy for enriching anthracene. The highest total content of the three types of AVN was obtained on the fifth day of germination. The germination treatment significantly increased the contents of the three types of AVNs, but the degrees of enrichment of AVN-B and AVN-C were higher than that of AVN-A, and the contents of the three types of AVNs differed with the oat varieties. AVN exhibits good antisensitivity and antioxidant activities, as well as a dose–response relationship with the content in oats. Further, a certain relationship existed between the antisensitivity and antioxidant activities.

## Figures and Tables

**Table 1:** Mass spectrometric conditions of the three anthranilamide.

Name	Molecular Formula	CAS Number	Molecular Structure	Precursor Ion (*m*/*z*)	Collision Energy (CE)	Retention Time (min)	Fragment Ion (*m*/*z*)
Avenanthramide A	C16H13NO5	108605-70-5	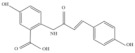	298.0721	30/40	6.8	254.0817/160.0394
Avenanthramide B	C17H15NO6	108605-69-2	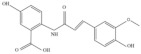	328.0821	30/40	6.83	284.0923/268.0611
Avenanthramide C	C16H13NO6	116764-15-9	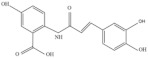	314.0665	20/30	6.66	178.0137/135.0441

**Table 2:** Optimum chromatographic conditions of the three main anthranilamide species.

Time (min)	Flow (mL/min)	B (%)	Injection Volume (µL)
0	0.45	2	5
3.5	0.45	2	5
7.0	0.45	98	5
7.5	0.45	98	5
8.0	0.45	2	5

**Table 3:** Linear equations and LODs and LOQs of the three anthranilamides.

Anthracamide Type	Ranger (μg/L)	Linear Equations	Coefficient (R)	LOD (μg/L)	LOQ (μg/L)
AVN-A	1–2000	y = 0.9654x + 5.5375	0.9989	2.2	6.6
AVN-B	1–2000	y = 0.9737x − 2.9207	0.9968	1.7	5.1
AVN-C	1–2000	y = 0.9713x − 2.5372	0.9971	3.7	11.1

**Table 4:** Recovery and precision of anthracene amide that was added to the sprouted oats.

	0.5 μg/L			1 μg/L			1.5 μg/L		
	Recovery (%)	Inter RSD (%)	Inter RSD (%)	Recovery (%)	Inter RSD (%)	Inter RSD (%)	Recovery (%)	Inter RSD (%)	Inter RSD (%)
AVN-A	109.5	4.8	5.9	112.9	8.4	8.3	113.2	5.1	10.1
AVN-B	103.9	5.4	2.7	115.9	3.9	6.4	111.6	10.2	17.1
AVN-C	100.4	4.7	4.2	110.2	4.3	6.6	118	8.8	12.8

**Table 5:** Changes in the AVN contents of oat after germination.

Germination Time	Zaohau Varieties			Bayou Varieties		
(d)	AVN-A (μg/g)	AVN-B (μg/g)	AVN-C (μg/g)	AVN-A (μg/g)	AVN-B (μg/g)	AVN-C (μg/g)
0	2.98 ± 0.24	1.63 ± 0.18	3.36 ± 0.27	7.58 ± 0.45	5.41 ± 0.19	5.29 ± 0.89
1	36.31 ± 2.49	34.09 ± 2.36	30.15 ± 1.99	18.37 ± 0.88	17.79 ± 0.74	9.25 ± 0.54
2	41.39 ± 2.94	39.36 ± 2.08	29.67 ± 1.28	19.51 ± 1.64	37.12 ± 1.68	20.91 ± 1.55
3	38.97 ± 1.51	49.22 ± 1.51	37.65 ± 2.14	34.76 ± 1.78	34.45 ± 2.18	27.14 ± 1.92
4	43.14 ± 1.69	51.53 ± 1.75	49.21 ± 2.64	35.42 ± 3.81	40.08 ± 3.37	28.22 ± 1.23
5	49.44 ± 2.12	53.46 ± 2.17	50.61 ± 1.79	39.24 ± 1.94	50.46 ± 2.05	36.6 ± 3.16
6	43.21 ± 4.11	50.35 ± 3.58	54.23 ± 3.77	39.73 ± 1.50	33.13 ± 1.71	36.57 ± 2.55
7	38.03 ± 3.04	39.58 ± 1.33	55.43 ± 2.48	36.28 ± 1.07	35.40 ± 1.22	36.86 ± 1.35
8	39.34 ± 2.80	47.26 ± 2.33	60.75 ± 2.40	33.92 ± 2.82	27.76 ± 1.16	41.04 ± 2.06

## Data Availability

Not applicable. Samples are not available from the authors.

## Supplementary Materials

The following supporting information can be downloaded at: https://www.mdpi.com/article/10.3390/molecules27196167/s1. Figure A: Optimize collision energy—Anthracem ion mass spectrometry.
