# Supplementary figures and images for: Effect of Germination on the Avenanthramide Content of Oats and Their in Vitro Antisensitivity Activities
Source: Molecules. 2022 Sep 20;27(19):6167. doi: 10.3390/molecules27196167 (PMC9573532; doi:10.3390/molecules27196167)

# Supplementary Figure S1

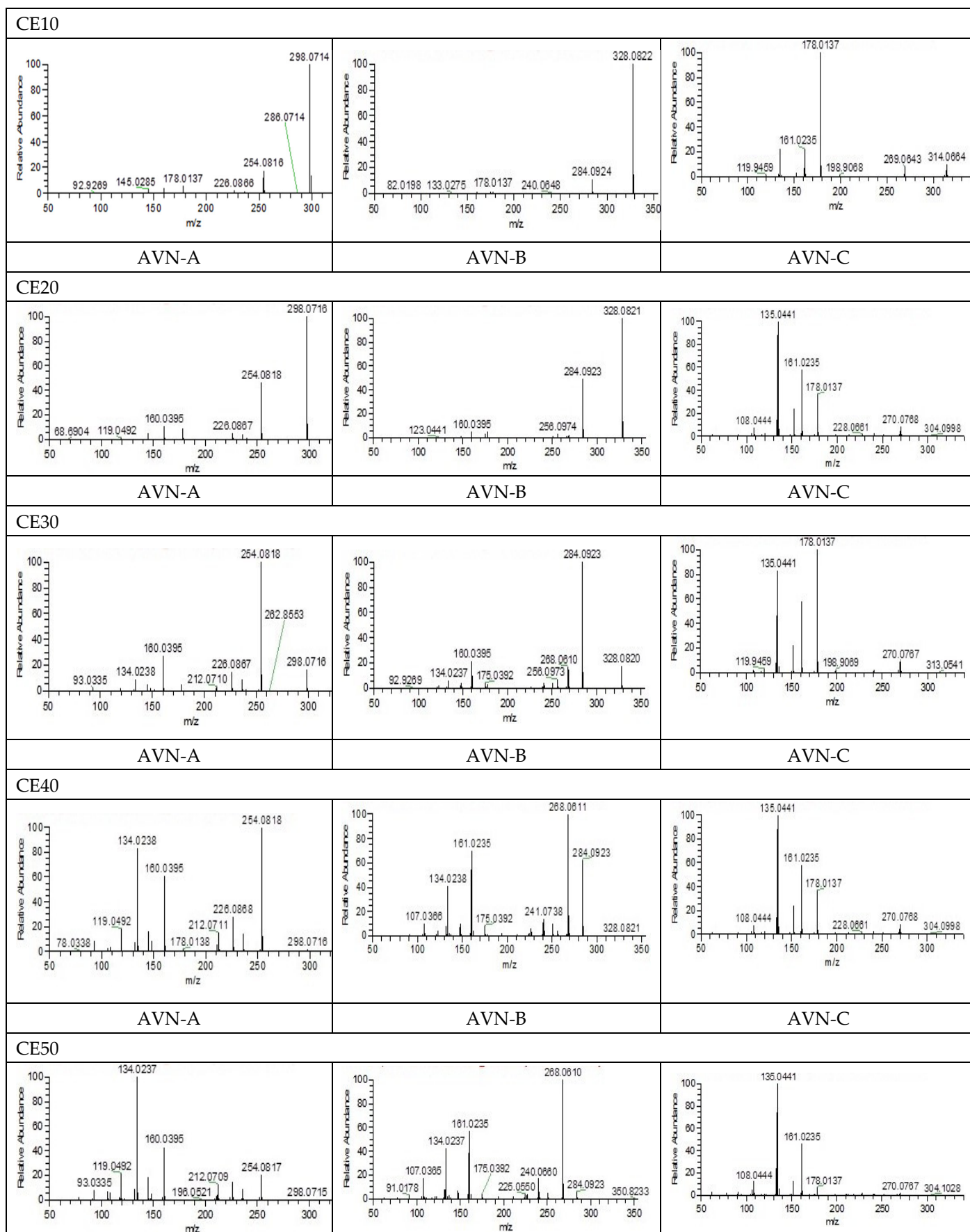

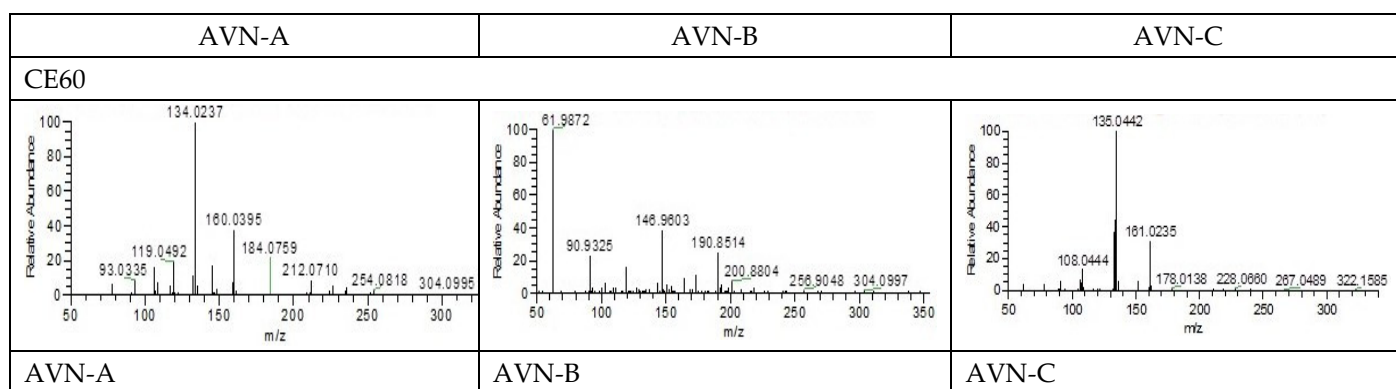

**Figure S1.** Optimize collision energy—Anthracem ion mass spectrometry.

Supplement: Supplementary file 1 [file molecules-27-06167-s001.zip › molecules-1868497-supplementary.pdf]
